# [3,3′-Dimesityl-1,1′-(4,5:16,17-dibenzo-3,6,9,12,15,18-hexa­oxaeicosane-1,20-di­yl)diimidazolin-2-yl­idene]dithio­cyanato­palladium(II)

**DOI:** 10.1107/S1600536809014615

**Published:** 2009-04-25

**Authors:** Xiao-Qin Zhang, Mei-Ming Luo

**Affiliations:** a Key Laboratory of Green Chemistry and Technology of the Ministry of Education, College of Chemistry, Sichuan University, Chengdu 610064, People’s Republic of China

## Abstract

The coordination geometry of the Pd atom in the title compound, [Pd(SCN)_2_(C_46_H_54_N_4_O_6_)], is approximately square-planar. The N-heterocyclic carbene (NHC) metallacrown ether ligand binds to the Pd atom in a *trans* orientation through the carbene C atoms of the two imidazole rings and generates a 25-membered chelate ring. Two mutually *trans* S-bound thio­cyanate ligands complete the coordination.

## Related literature

For *N*-heterocyclic carbene ligands and their complexes, see: Herrmann (2002 ▶); Hahn & Jahnke (2008 ▶). For details of bis-phosphine polyether ligands, see: Alcock *et al.* (1976 ▶); Powell *et al.* (1981 ▶); Gray *et al.* (1995 ▶). For mixed NHC metallacrown ether ligands, see: Nielsen *et al.* (2003 ▶); Liu *et al.* (2007 ▶); Wang *et al.* (2005 ▶). For the use of Pd–NHC complexes in catalysis, see: Herrmann *et al.* (2002 ▶); Kantchev *et al.* (2007 ▶). For the synthesis of the ligand precursor, see: Pedersen (1967 ▶); Haque & Rasmussen (1994 ▶).
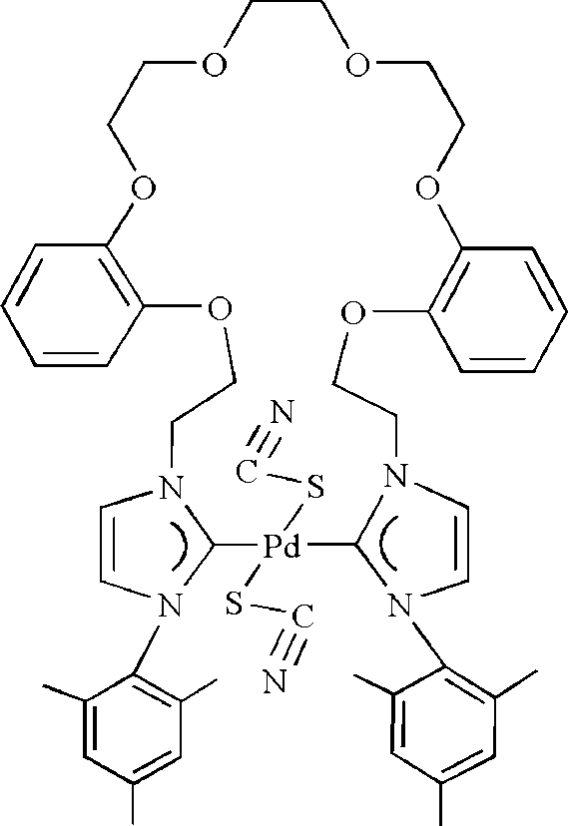


## Experimental

### 

#### Crystal data

[Pd(NCS)_2_(C_46_H_54_N_4_O_6_)]
*M* *_r_* = 981.49
Monoclinic, 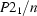

*a* = 14.143 (5) Å
*b* = 19.803 (4) Å
*c* = 17.101 (3) Å
β = 97.13 (2)°
*V* = 4753 (2) Å^3^
*Z* = 4
Mo *K*α radiation
μ = 0.53 mm^−1^
*T* = 288 K
0.46 × 0.42 × 0.40 mm

#### Data collection

Enraf–Nonius CAD-4 diffractometer
Absorption correction: spherical (Farrugia, 1999 ▶) *T*_min_ = 0.927, *T*_max_ = 0.936
9805 measured reflections
8672 independent reflections
4983 reflections with *I* > 2σ(*I*)
*R*_int_ = 0.003
3 standard reflections every 300 reflections intensity decay: 1.2%

#### Refinement

*R*[*F* ^2^ > 2σ(*F* ^2^)] = 0.066
*wR*(*F* ^2^) = 0.218
*S* = 1.05
8672 reflections
566 parameters
61 restraints
H-atom parameters constrained
Δρ_max_ = 0.78 e Å^−3^
Δρ_min_ = −1.31 e Å^−3^

### 

Data collection: *DIFRAC* (Gabe *et al.*, 1993 ▶); cell refinement: *DIFRAC*; data reduction: *DIFRAC*; program(s) used to solve structure: *SHELXS97* (Sheldrick, 2008 ▶); program(s) used to refine structure: *SHELXL97* (Sheldrick, 2008 ▶); molecular graphics: *ORTEP-3 for Windows* (Farrugia, 1997 ▶); software used to prepare material for publication: *SHELXL97*.

## Supplementary Material

Crystal structure: contains datablocks I, global. DOI: 10.1107/S1600536809014615/sj2617sup1.cif

Structure factors: contains datablocks I. DOI: 10.1107/S1600536809014615/sj2617Isup2.hkl

Additional supplementary materials:  crystallographic information; 3D view; checkCIF report

## Figures and Tables

**Table:** 

Pd1—C1	2.040 (6)
Pd1—C35	2.041 (6)
Pd1—S2	2.3211 (18)
Pd1—S1	2.3237 (19)

**Table:** 

C1—Pd1—C35	178.6 (3)
C1—Pd1—S2	85.12 (17)
C35—Pd1—S2	95.47 (19)
C1—Pd1—S1	94.03 (17)
C35—Pd1—S1	85.31 (19)
S2—Pd1—S1	176.52 (8)

**Fig. 1.**
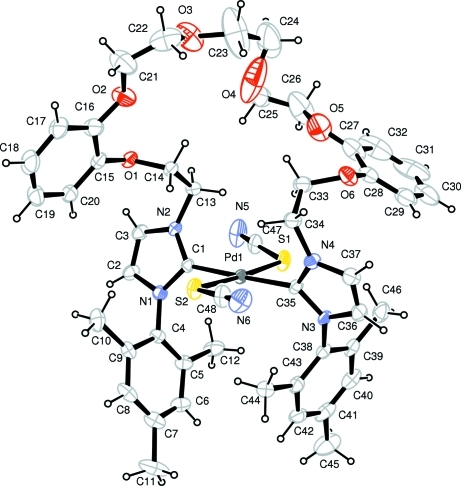
The molecular structure of the title compound with displacement ellipsoids drawn at the 30% probability level.

## supplementary crystallographic information

### Comment

N-Heterocyclic carbene (NHC) ligands have been found to be interesting substitutes for phosphine ligands and are employed with considerable success in coordination chemistry and various catalytic transformations (Herrmann *et al.*, 2002; Hahn & Jahnke, 2008). Much interest has been devoted to the chemistry of metallacrown ethers formed by the chelation of bis(phosphorus-donor)polyether ligands to transition metals for a number of years (Alcock *et al.*,1976; Powell *et al.*, 1981; Gray, 1995). Studies on these metallacrown ethers have shown that they can bind hard metal cations and such hard–soft bimetallic complexes are of interest as catalysts for organic reactions. Substitution of phosphine donors by NHCs has led to several examples of mixed NHC metallacrown ether ligands (Nielsen *et al.*, 2003; Liu *et al.*, 2007; Wang *et al.*, 2005). Pd–NHC complexes are known to catalyze a wide range of useful cross-coupling reactions (Herrmann, 2002; Kantchev *et al.*, 2007). However, we were surprised that no Pd–NHC metallacrown ether complexes have been described to date and we present here the first synthesis and crystal structure of the title Pd–NHC metallacrown ether complex.

In the title compound (Fig. 1), a 25-membered Pd–NHC metallacrown ether complex adopting a *trans*-conformation is formed by a bidentate chelate bis(carbene) ligand with a long flexible linkage and a Pd(II). The coordination geometry at Pd is approximately square planar with the C1–Pd–C35 angle of 178.6 (3)° and the S1–Pd–S2 angle of 178.6 (3)°. The two benzene rings linked with ether oxygen atoms form a dihedral angle of 81.20°. The dihedral angle of the NHC rings is 39.38°.

### Experimental

A mixture of 1,20-di(1-mesitylimidazolium)-4,5,16,17-dibenzo-3,6,9,12,15,18-hexaoxaeicosane dichloride (83.2 mg, 0.10 mmol) prepared by literature procedures (Pedersen, 1967; Haque & Rasmussen, 1994) and silver(I) oxide (27.6 mg, 0.12 mmol) in 5 ml of CH_2_Cl_2_ was stirred at room temperature for 2 h. The reaction mixture was filtered and washed with CH_2_Cl_2 _(5 ml × 2). The combined filtrate was reduced to 5 ml under vacuum. [PdCl_2_(MeCN)_2_] (25.8 mg, 0.10 mmol) in CH_2_Cl_2_ (3 ml) was added to the resulting solution and stirred at room temperature for 2 h, then KSCN (97 mg, 1 mmol) was added and stirred over night. The reaction mixture was filtered and washed with CH_2_Cl_2_ (5 ml × 2). The combined solution was evaporated under reduced pressure to leave a raw product, which was purified by flash chromatography on silica gel (CH_2_Cl_2_) to give a yellow solid. Single crystals suitable for X-ray diffraction were obtained at ambient temperature by slow evaporation of an Et_2_O solution over a period of several days.

### Refinement

All H atom were positioned geometrically with C—H = 0.93 Å (aromatic) or 0.96 Å (methyl) and refined using a riding model with 1.5 *U*_eq_(C) for methyl and *U*_iso_(H) = 1.2 *U*_eq_(C) for others.

### Crystal data

**Table:**

[Pd(NCS)_2_(C_46_H_54_N_4_O_6_)]	*F*(000) = 2040
*M**_r_* = 981.49	*D*_x_ = 1.372 Mg m^−^^3^
Monoclinic, *P*2_1_/*n*	Mo *K*α radiation, λ = 0.71073 Å
Hall symbol: -P 2yn	Cell parameters from 40 reflections
*a* = 14.143 (5) Å	θ = 5.0–7.8°
*b* = 19.803 (4) Å	µ = 0.53 mm^−^^1^
*c* = 17.101 (3) Å	*T* = 288 K
β = 97.13 (2)°	Block, colourless
*V* = 4753 (2) Å^3^	0.46 × 0.42 × 0.40 mm
*Z* = 4	

### Data collection

**Table:**

Enraf–Nonius CAD-4 diffractometer	4983 reflections with *I* > 2σ(*I*)
Radiation source: fine-focus sealed tube	*R*_int_ = 0.003
graphite	θ_max_ = 25.5°, θ_min_ = 1.8°
ω/2θ scans	*h* = −17→16
Absorption correction: for a sphere (Farrugia, 1999)	*k* = 0→24
*T*_min_ = 0.927, *T*_max_ = 0.936	*l* = −7→20
9805 measured reflections	3 standard reflections every 300 reflections
8672 independent reflections	intensity decay: 1.2%

### Refinement

**Table:**

Refinement on *F*^2^	Primary atom site location: structure-invariant direct methods
Least-squares matrix: full	Secondary atom site location: difference Fourier map
*R*[*F*^2^ > 2σ(*F*^2^)] = 0.066	Hydrogen site location: inferred from neighbouring sites
*wR*(*F*^2^) = 0.218	H-atom parameters constrained
*S* = 1.05	*w* = 1/[σ^2^(*F*_o_^2^) + (0.1301*P*)^2^] where *P* = (*F*_o_^2^ + 2*F*_c_^2^)/3
8672 reflections	(Δ/σ)_max_ < 0.001
566 parameters	Δρ_max_ = 0.78 e Å^−^^3^
61 restraints	Δρ_min_ = −1.31 e Å^−^^3^

### Special details

**Table:**

Geometry. All e.s.d.'s (except the e.s.d. in the dihedral angle between two l.s. planes) are estimated using the full covariance matrix. The cell e.s.d.'s are taken into account individually in the estimation of e.s.d.'s in distances, angles and torsion angles; correlations between e.s.d.'s in cell parameters are only used when they are defined by crystal symmetry. An approximate (isotropic) treatment of cell e.s.d.'s is used for estimating e.s.d.'s involving l.s. planes.
Refinement. Refinement of *F*^2^ against ALL reflections. The weighted *R*-factor *wR* and goodness of fit *S* are based on *F*^2^, conventional *R*-factors *R* are based on *F*, with *F* set to zero for negative *F*^2^. The threshold expression of *F*^2^ > σ(*F*^2^) is used only for calculating *R*-factors(gt) *etc*. and is not relevant to the choice of reflections for refinement. *R*-factors based on *F*^2^ are statistically about twice as large as those based on *F*, and *R*- factors based on ALL data will be even larger.

### Fractional atomic coordinates and isotropic or equivalent isotropic displacement parameters (Å^2^)

**Table:**

	*x*	*y*	*z*	*U*_iso_*/*U*_eq_	
Pd1	0.57564 (3)	0.29052 (2)	0.23443 (3)	0.04415 (19)	
S1	0.64646 (14)	0.18450 (9)	0.24534 (15)	0.0721 (6)	
S2	0.50579 (14)	0.39669 (9)	0.23181 (14)	0.0689 (6)	
O1	0.7414 (3)	0.4138 (2)	0.4806 (3)	0.0610 (12)	
O2	0.6397 (6)	0.4445 (3)	0.5970 (4)	0.122 (3)	
O3	0.5875 (5)	0.3396 (5)	0.6878 (7)	0.162 (4)	
O4	0.6080 (11)	0.2342 (6)	0.5779 (10)	0.215 (6)	
O5	0.5559 (7)	0.1325 (5)	0.4902 (6)	0.159 (4)	
O6	0.3911 (4)	0.1529 (3)	0.3957 (4)	0.0852 (17)	
N1	0.7502 (4)	0.3790 (3)	0.2186 (3)	0.0541 (14)	
N2	0.7516 (4)	0.3414 (3)	0.3370 (3)	0.0482 (13)	
N3	0.4200 (4)	0.2032 (3)	0.1410 (3)	0.0505 (13)	
N4	0.3804 (4)	0.2329 (3)	0.2526 (3)	0.0574 (14)	
N5	0.8391 (6)	0.1955 (4)	0.3031 (7)	0.125 (4)	
N6	0.3073 (6)	0.3895 (4)	0.2113 (5)	0.103 (3)	
C1	0.7006 (4)	0.3409 (3)	0.2651 (4)	0.0461 (15)	
C2	0.8339 (5)	0.4017 (4)	0.2629 (4)	0.066 (2)	
H2	0.8808	0.4282	0.2446	0.079*	
C3	0.8342 (5)	0.3784 (4)	0.3365 (4)	0.0634 (19)	
H3	0.8809	0.3857	0.3790	0.076*	
C4	0.7200 (5)	0.3999 (4)	0.1393 (4)	0.0546 (17)	
C5	0.7266 (5)	0.3548 (4)	0.0775 (4)	0.064 (2)	
C6	0.6955 (6)	0.3780 (5)	0.0023 (5)	0.077 (2)	
H6	0.7016	0.3494	−0.0400	0.093*	
C7	0.6575 (7)	0.4386 (6)	−0.0131 (6)	0.092 (3)	
C8	0.6537 (7)	0.4832 (5)	0.0501 (6)	0.090 (3)	
H8	0.6293	0.5264	0.0403	0.108*	
C9	0.6852 (5)	0.4645 (4)	0.1264 (5)	0.066 (2)	
C10	0.6829 (7)	0.5139 (4)	0.1941 (6)	0.096 (3)	
H10A	0.7457	0.5316	0.2091	0.144*	
H10B	0.6616	0.4910	0.2382	0.144*	
H10C	0.6401	0.5502	0.1778	0.144*	
C11	0.6202 (10)	0.4598 (7)	−0.0983 (6)	0.158 (6)	
H11A	0.6583	0.4389	−0.1342	0.237*	
H11B	0.6239	0.5080	−0.1030	0.237*	
H11C	0.5551	0.4457	−0.1106	0.237*	
C12	0.7657 (7)	0.2847 (4)	0.0895 (5)	0.087 (3)	
H12A	0.7997	0.2808	0.1415	0.131*	
H12B	0.8083	0.2755	0.0512	0.131*	
H12C	0.7143	0.2527	0.0834	0.131*	
C13	0.7204 (5)	0.3113 (4)	0.4082 (4)	0.0572 (17)	
H13A	0.7750	0.2935	0.4418	0.069*	
H13B	0.6767	0.2744	0.3936	0.069*	
C14	0.6710 (5)	0.3653 (4)	0.4527 (4)	0.0599 (18)	
H14A	0.6202	0.3865	0.4179	0.072*	
H14B	0.6438	0.3452	0.4965	0.072*	
C15	0.7069 (5)	0.4784 (4)	0.4864 (5)	0.065 (2)	
C16	0.6561 (7)	0.4964 (5)	0.5486 (6)	0.088 (3)	
C17	0.6275 (9)	0.5624 (5)	0.5563 (6)	0.107 (4)	
H17	0.5935	0.5749	0.5972	0.129*	
C18	0.6500 (10)	0.6088 (5)	0.5031 (8)	0.121 (4)	
H18	0.6318	0.6535	0.5088	0.145*	
C19	0.6984 (8)	0.5921 (5)	0.4413 (7)	0.101 (3)	
H19	0.7113	0.6246	0.4049	0.121*	
C20	0.7274 (6)	0.5269 (4)	0.4341 (5)	0.077 (2)	
H20	0.7616	0.5153	0.3930	0.092*	
C21	0.5876 (13)	0.4567 (6)	0.6570 (8)	0.169 (7)	
H21A	0.5407	0.4911	0.6399	0.203*	
H21B	0.6298	0.4754	0.7007	0.203*	
C22	0.5373 (10)	0.3984 (7)	0.6865 (8)	0.147 (6)	
H22A	0.5233	0.4083	0.7394	0.176*	
H22B	0.4771	0.3924	0.6533	0.176*	
C23	0.5281 (11)	0.2759 (8)	0.6820 (15)	0.217 (8)	
H23A	0.4685	0.2861	0.6498	0.261*	
H23B	0.5128	0.2656	0.7345	0.261*	
C24	0.5684 (16)	0.2106 (8)	0.6487 (11)	0.204 (8)	
H24A	0.6176	0.1908	0.6864	0.245*	
H24B	0.5184	0.1775	0.6353	0.245*	
C25	0.6713 (14)	0.1847 (9)	0.5687 (13)	0.204 (8)	
H25A	0.6986	0.1710	0.6211	0.245*	
H25B	0.7224	0.2051	0.5440	0.245*	
C26	0.6442 (11)	0.1235 (9)	0.5252 (12)	0.189 (7)	
H26A	0.6871	0.1151	0.4862	0.227*	
H26B	0.6467	0.0851	0.5607	0.227*	
C27	0.5279 (6)	0.0829 (5)	0.4391 (5)	0.123 (4)	
C28	0.4429 (5)	0.0943 (3)	0.3907 (4)	0.084 (3)	
C29	0.4052 (6)	0.0443 (5)	0.3388 (4)	0.116 (4)	
H29	0.3483	0.0519	0.3065	0.139*	
C30	0.4524 (9)	−0.0169 (4)	0.3353 (5)	0.157 (7)	
H30	0.4272	−0.0503	0.3006	0.189*	
C31	0.5374 (9)	−0.0283 (4)	0.3836 (7)	0.196 (10)	
H31	0.5690	−0.0692	0.3813	0.235*	
C32	0.5751 (5)	0.0217 (6)	0.4355 (6)	0.172 (8)	
H32	0.6320	0.0141	0.4679	0.206*	
C33	0.4426 (6)	0.2152 (4)	0.3916 (5)	0.077 (2)	
H33A	0.5048	0.2066	0.3753	0.092*	
H33B	0.4514	0.2369	0.4428	0.092*	
C34	0.3849 (6)	0.2597 (4)	0.3325 (4)	0.068 (2)	
H34A	0.3208	0.2639	0.3465	0.082*	
H34B	0.4130	0.3044	0.3341	0.082*	
C35	0.4510 (4)	0.2390 (3)	0.2064 (4)	0.0489 (15)	
C36	0.3311 (5)	0.1766 (4)	0.1479 (5)	0.072 (2)	
H36	0.2948	0.1501	0.1106	0.086*	
C37	0.3067 (5)	0.1957 (4)	0.2169 (5)	0.073 (2)	
H37	0.2501	0.1856	0.2369	0.088*	
C38	0.4700 (5)	0.1907 (4)	0.0744 (4)	0.0552 (17)	
C39	0.5038 (5)	0.1248 (4)	0.0659 (5)	0.070 (2)	
C40	0.5501 (6)	0.1117 (6)	0.0002 (6)	0.093 (3)	
H40	0.5730	0.0685	−0.0070	0.111*	
C41	0.5629 (6)	0.1605 (7)	−0.0543 (6)	0.096 (3)	
C42	0.5268 (6)	0.2250 (5)	−0.0448 (5)	0.078 (3)	
H42	0.5340	0.2580	−0.0823	0.094*	
C43	0.4801 (5)	0.2409 (4)	0.0200 (5)	0.069 (2)	
C44	0.4422 (7)	0.3107 (4)	0.0287 (5)	0.083 (2)	
H44A	0.3959	0.3101	0.0653	0.124*	
H44B	0.4128	0.3266	−0.0216	0.124*	
H44C	0.4936	0.3404	0.0479	0.124*	
C45	0.6116 (8)	0.1436 (8)	−0.1273 (7)	0.148 (5)	
H45A	0.6633	0.1129	−0.1129	0.222*	
H45B	0.6357	0.1843	−0.1480	0.222*	
H45C	0.5662	0.1231	−0.1666	0.222*	
C46	0.4916 (7)	0.0701 (4)	0.1243 (6)	0.096 (3)	
H46A	0.4261	0.0561	0.1188	0.143*	
H46B	0.5097	0.0868	0.1767	0.143*	
H46C	0.5311	0.0323	0.1148	0.143*	
C47	0.7609 (6)	0.1926 (4)	0.2804 (6)	0.072 (2)	
C48	0.3883 (6)	0.3909 (4)	0.2185 (6)	0.077 (2)	

### Atomic displacement parameters (Å^2^)

**Table:**

	*U*^11^	*U*^22^	*U*^33^	*U*^12^	*U*^13^	*U*^23^
Pd1	0.0448 (3)	0.0386 (3)	0.0478 (3)	0.0031 (2)	0.0011 (2)	−0.0046 (2)
S1	0.0625 (11)	0.0423 (9)	0.1069 (17)	0.0070 (9)	−0.0080 (11)	−0.0124 (11)
S2	0.0598 (11)	0.0452 (10)	0.0998 (16)	0.0093 (8)	0.0020 (11)	−0.0054 (10)
O1	0.061 (3)	0.060 (3)	0.058 (3)	0.003 (2)	−0.007 (2)	−0.014 (2)
O2	0.202 (8)	0.081 (5)	0.097 (5)	0.047 (5)	0.074 (5)	0.003 (4)
O3	0.082 (5)	0.181 (9)	0.233 (10)	0.009 (6)	0.061 (6)	0.043 (8)
O4	0.212 (13)	0.141 (10)	0.275 (15)	−0.039 (9)	−0.034 (11)	0.012 (11)
O5	0.101 (6)	0.173 (9)	0.191 (10)	−0.005 (6)	−0.031 (6)	0.053 (8)
O6	0.079 (4)	0.089 (4)	0.090 (4)	−0.008 (3)	0.017 (3)	0.022 (3)
N1	0.053 (3)	0.058 (3)	0.053 (3)	−0.004 (3)	0.012 (3)	−0.003 (3)
N2	0.046 (3)	0.050 (3)	0.047 (3)	−0.002 (2)	0.000 (2)	−0.010 (3)
N3	0.052 (3)	0.050 (3)	0.045 (3)	−0.007 (3)	−0.011 (2)	−0.007 (3)
N4	0.048 (3)	0.069 (4)	0.054 (3)	0.000 (3)	0.004 (3)	−0.006 (3)
N5	0.073 (5)	0.075 (5)	0.215 (12)	0.017 (4)	−0.031 (6)	−0.011 (6)
N6	0.070 (5)	0.113 (7)	0.119 (7)	0.017 (5)	−0.014 (5)	−0.009 (5)
C1	0.052 (4)	0.036 (3)	0.050 (4)	0.004 (3)	0.004 (3)	−0.003 (3)
C2	0.044 (4)	0.091 (6)	0.061 (5)	−0.010 (4)	0.002 (3)	−0.006 (4)
C3	0.048 (4)	0.080 (5)	0.060 (5)	−0.003 (4)	−0.001 (3)	−0.012 (4)
C4	0.050 (4)	0.060 (4)	0.054 (4)	0.001 (3)	0.009 (3)	0.009 (3)
C5	0.065 (5)	0.073 (5)	0.057 (5)	0.012 (4)	0.017 (4)	0.000 (4)
C6	0.081 (6)	0.099 (7)	0.053 (5)	0.007 (5)	0.013 (4)	−0.001 (5)
C7	0.092 (7)	0.105 (8)	0.082 (6)	0.009 (6)	0.021 (5)	0.031 (6)
C8	0.092 (6)	0.077 (6)	0.105 (7)	0.013 (5)	0.026 (6)	0.044 (6)
C9	0.068 (5)	0.054 (4)	0.079 (5)	−0.004 (4)	0.017 (4)	0.014 (4)
C10	0.109 (7)	0.055 (5)	0.128 (8)	−0.004 (5)	0.027 (6)	−0.012 (5)
C11	0.170 (12)	0.215 (15)	0.088 (8)	0.022 (11)	0.016 (8)	0.083 (9)
C12	0.109 (7)	0.093 (6)	0.060 (5)	0.039 (5)	0.013 (5)	−0.014 (5)
C13	0.062 (4)	0.061 (4)	0.047 (4)	−0.004 (3)	0.004 (3)	−0.001 (3)
C14	0.065 (4)	0.065 (4)	0.050 (4)	−0.008 (4)	0.008 (3)	−0.002 (4)
C15	0.067 (5)	0.063 (5)	0.061 (5)	0.001 (4)	−0.008 (4)	−0.016 (4)
C16	0.109 (7)	0.078 (6)	0.077 (6)	0.021 (5)	0.013 (5)	−0.001 (5)
C17	0.167 (11)	0.059 (6)	0.097 (7)	0.024 (6)	0.021 (7)	−0.023 (5)
C18	0.162 (12)	0.064 (6)	0.133 (10)	0.014 (7)	0.000 (8)	−0.012 (7)
C19	0.125 (9)	0.061 (6)	0.114 (8)	−0.008 (6)	−0.002 (6)	0.011 (5)
C20	0.085 (6)	0.070 (5)	0.072 (5)	−0.012 (4)	−0.005 (5)	−0.002 (5)
C21	0.28 (2)	0.100 (9)	0.153 (12)	0.040 (11)	0.133 (13)	0.008 (9)
C22	0.132 (11)	0.205 (15)	0.105 (9)	0.057 (11)	0.020 (8)	−0.022 (10)
C23	0.116 (11)	0.191 (16)	0.35 (2)	−0.016 (12)	0.048 (14)	−0.012 (17)
C24	0.29 (2)	0.102 (11)	0.202 (16)	−0.047 (12)	−0.037 (14)	−0.038 (11)
C25	0.185 (16)	0.164 (15)	0.241 (19)	0.015 (14)	−0.060 (14)	0.056 (15)
C26	0.099 (10)	0.193 (16)	0.265 (19)	−0.028 (10)	−0.019 (11)	0.066 (15)
C27	0.087 (8)	0.135 (11)	0.149 (12)	0.011 (8)	0.022 (8)	0.062 (9)
C28	0.094 (7)	0.086 (6)	0.077 (6)	0.006 (5)	0.032 (5)	0.023 (5)
C29	0.171 (11)	0.093 (8)	0.091 (8)	−0.002 (8)	0.048 (8)	0.016 (7)
C30	0.27 (2)	0.095 (10)	0.124 (11)	0.002 (11)	0.109 (13)	0.016 (8)
C31	0.27 (2)	0.198 (19)	0.155 (15)	0.096 (17)	0.153 (16)	0.059 (14)
C32	0.151 (12)	0.198 (16)	0.188 (16)	0.093 (13)	0.101 (12)	0.088 (14)
C33	0.079 (5)	0.092 (6)	0.059 (5)	−0.005 (4)	0.006 (4)	0.004 (4)
C34	0.074 (5)	0.069 (5)	0.063 (5)	0.000 (4)	0.011 (4)	0.001 (4)
C35	0.046 (4)	0.047 (3)	0.051 (4)	0.002 (3)	−0.006 (3)	−0.005 (3)
C36	0.054 (4)	0.083 (6)	0.072 (6)	−0.016 (4)	−0.016 (4)	0.006 (5)
C37	0.045 (4)	0.095 (6)	0.079 (6)	−0.007 (4)	0.003 (4)	0.016 (5)
C38	0.044 (4)	0.066 (4)	0.052 (4)	−0.004 (3)	−0.004 (3)	−0.010 (3)
C39	0.060 (4)	0.067 (5)	0.077 (5)	−0.002 (4)	−0.011 (4)	−0.019 (4)
C40	0.072 (6)	0.105 (7)	0.097 (7)	0.010 (5)	−0.006 (5)	−0.046 (5)
C41	0.054 (5)	0.151 (10)	0.082 (7)	−0.010 (6)	0.002 (5)	−0.050 (6)
C42	0.070 (5)	0.113 (7)	0.049 (5)	−0.021 (5)	−0.002 (4)	−0.001 (5)
C43	0.062 (5)	0.076 (5)	0.069 (5)	−0.015 (4)	0.006 (4)	−0.013 (4)
C44	0.100 (6)	0.084 (5)	0.063 (5)	−0.004 (5)	0.004 (5)	0.007 (5)
C45	0.107 (8)	0.242 (16)	0.103 (9)	−0.004 (10)	0.042 (7)	−0.054 (10)
C46	0.111 (7)	0.058 (5)	0.111 (8)	0.004 (5)	−0.014 (6)	−0.013 (5)
C47	0.068 (5)	0.041 (4)	0.103 (7)	0.015 (4)	−0.003 (5)	−0.007 (4)
C48	0.063 (5)	0.057 (5)	0.112 (7)	0.020 (4)	0.009 (5)	−0.003 (5)

### Geometric parameters (Å, °)

**Table:**

Pd1—C1	2.040 (6)	C16—C17	1.379 (12)
Pd1—C35	2.041 (6)	C17—C18	1.358 (14)
Pd1—S2	2.3211 (18)	C17—H17	0.9300
Pd1—S1	2.3237 (19)	C18—C19	1.369 (15)
S1—C47	1.662 (9)	C18—H18	0.9300
S2—C48	1.653 (9)	C19—C20	1.364 (13)
O1—C15	1.377 (9)	C19—H19	0.9300
O1—C14	1.422 (8)	C20—H20	0.9300
O2—C21	1.357 (12)	C21—C22	1.477 (14)
O2—C16	1.359 (11)	C21—H21A	0.9700
O3—C22	1.362 (12)	C21—H21B	0.9700
O3—C23	1.513 (14)	C22—H22A	0.9700
O4—C25	1.350 (15)	C22—H22B	0.9700
O4—C24	1.472 (15)	C23—C24	1.549 (15)
O5—C26	1.329 (15)	C23—H23A	0.9700
O5—C27	1.341 (11)	C23—H23B	0.9700
O6—C28	1.381 (8)	C24—H24A	0.9700
O6—C33	1.439 (9)	C24—H24B	0.9700
N1—C1	1.354 (8)	C25—C26	1.449 (15)
N1—C2	1.398 (9)	C25—H25A	0.9700
N1—C4	1.431 (8)	C25—H25B	0.9700
N2—C1	1.347 (8)	C26—H26A	0.9700
N2—C3	1.379 (9)	C26—H26B	0.9700
N2—C13	1.472 (8)	C27—C28	1.3900
N3—C35	1.351 (8)	C27—C32	1.3900
N3—C36	1.381 (9)	C28—C29	1.3900
N3—C38	1.435 (8)	C29—C30	1.3900
N4—C35	1.355 (8)	C29—H29	0.9300
N4—C37	1.358 (9)	C30—C31	1.3900
N4—C34	1.459 (9)	C30—H30	0.9300
N5—C47	1.128 (10)	C31—C32	1.3900
N6—C48	1.137 (10)	C31—H31	0.9300
C2—C3	1.342 (10)	C32—H32	0.9300
C2—H2	0.9300	C33—C34	1.503 (10)
C3—H3	0.9300	C33—H33A	0.9700
C4—C9	1.379 (10)	C33—H33B	0.9700
C4—C5	1.395 (10)	C34—H34A	0.9700
C5—C6	1.384 (10)	C34—H34B	0.9700
C5—C12	1.501 (11)	C36—C37	1.325 (11)
C6—C7	1.330 (12)	C36—H36	0.9300
C6—H6	0.9300	C37—H37	0.9300
C7—C8	1.402 (14)	C38—C43	1.382 (11)
C7—C11	1.544 (13)	C38—C39	1.404 (10)
C8—C9	1.375 (12)	C39—C40	1.393 (12)
C8—H8	0.9300	C39—C46	1.498 (12)
C9—C10	1.520 (12)	C40—C41	1.369 (14)
C10—H10A	0.9600	C40—H40	0.9300
C10—H10B	0.9600	C41—C42	1.392 (14)
C10—H10C	0.9600	C41—C45	1.536 (13)
C11—H11A	0.9600	C42—C43	1.394 (11)
C11—H11B	0.9600	C42—H42	0.9300
C11—H11C	0.9600	C43—C44	1.498 (12)
C12—H12A	0.9600	C44—H44A	0.9600
C12—H12B	0.9600	C44—H44B	0.9600
C12—H12C	0.9600	C44—H44C	0.9600
C13—C14	1.530 (9)	C45—H45A	0.9600
C13—H13A	0.9700	C45—H45B	0.9600
C13—H13B	0.9700	C45—H45C	0.9600
C14—H14A	0.9700	C46—H46A	0.9600
C14—H14B	0.9700	C46—H46B	0.9600
C15—C20	1.367 (11)	C46—H46C	0.9600
C15—C16	1.402 (12)		
			
C1—Pd1—C35	178.6 (3)	O3—C22—C21	113.7 (11)
C1—Pd1—S2	85.12 (17)	O3—C22—H22A	108.8
C35—Pd1—S2	95.47 (19)	C21—C22—H22A	108.8
C1—Pd1—S1	94.03 (17)	O3—C22—H22B	108.8
C35—Pd1—S1	85.31 (19)	C21—C22—H22B	108.8
S2—Pd1—S1	176.52 (8)	H22A—C22—H22B	107.7
C47—S1—Pd1	109.4 (3)	O3—C23—C24	119.6 (13)
C48—S2—Pd1	111.0 (3)	O3—C23—H23A	107.4
C15—O1—C14	114.3 (5)	C24—C23—H23A	107.4
C21—O2—C16	118.5 (8)	O3—C23—H23B	107.4
C22—O3—C23	115.4 (10)	C24—C23—H23B	107.4
C25—O4—C24	101.0 (16)	H23A—C23—H23B	107.0
C26—O5—C27	112.3 (13)	O4—C24—C23	103.4 (16)
C28—O6—C33	116.2 (6)	O4—C24—H24A	111.1
C1—N1—C2	109.1 (6)	C23—C24—H24A	111.1
C1—N1—C4	127.0 (6)	O4—C24—H24B	111.1
C2—N1—C4	123.6 (6)	C23—C24—H24B	111.1
C1—N2—C3	110.9 (6)	H24A—C24—H24B	109.0
C1—N2—C13	124.8 (5)	O4—C25—C26	122.0 (17)
C3—N2—C13	124.1 (6)	O4—C25—H25A	106.8
C35—N3—C36	109.6 (6)	C26—C25—H25A	106.8
C35—N3—C38	127.3 (5)	O4—C25—H25B	106.8
C36—N3—C38	123.0 (6)	C26—C25—H25B	106.8
C35—N4—C37	111.5 (6)	H25A—C25—H25B	106.7
C35—N4—C34	124.3 (6)	O5—C26—C25	107.0 (16)
C37—N4—C34	124.1 (6)	O5—C26—H26A	110.3
N2—C1—N1	105.9 (5)	C25—C26—H26A	110.3
N2—C1—Pd1	126.0 (5)	O5—C26—H26B	110.3
N1—C1—Pd1	128.1 (5)	C25—C26—H26B	110.3
C3—C2—N1	107.5 (6)	H26A—C26—H26B	108.6
C3—C2—H2	126.3	O5—C27—C28	115.7 (8)
N1—C2—H2	126.3	O5—C27—C32	124.2 (8)
C2—C3—N2	106.6 (6)	C28—C27—C32	120.0
C2—C3—H3	126.7	O6—C28—C29	118.2 (7)
N2—C3—H3	126.7	O6—C28—C27	121.7 (7)
C9—C4—C5	121.9 (7)	C29—C28—C27	120.0
C9—C4—N1	118.6 (7)	C28—C29—C30	120.0
C5—C4—N1	119.5 (6)	C28—C29—H29	120.0
C6—C5—C4	116.7 (7)	C30—C29—H29	120.0
C6—C5—C12	120.1 (7)	C29—C30—C31	120.0
C4—C5—C12	123.2 (7)	C29—C30—H30	120.0
C7—C6—C5	123.8 (9)	C31—C30—H30	120.0
C7—C6—H6	118.1	C30—C31—C32	120.0
C5—C6—H6	118.1	C30—C31—H31	120.0
C6—C7—C8	118.0 (9)	C32—C31—H31	120.0
C6—C7—C11	121.0 (11)	C31—C32—C27	120.0
C8—C7—C11	121.1 (10)	C31—C32—H32	120.0
C9—C8—C7	121.6 (8)	C27—C32—H32	120.0
C9—C8—H8	119.2	O6—C33—C34	107.4 (7)
C7—C8—H8	119.2	O6—C33—H33A	110.2
C8—C9—C4	118.0 (8)	C34—C33—H33A	110.2
C8—C9—C10	120.9 (8)	O6—C33—H33B	110.2
C4—C9—C10	121.1 (8)	C34—C33—H33B	110.2
C9—C10—H10A	109.5	H33A—C33—H33B	108.5
C9—C10—H10B	109.5	N4—C34—C33	111.9 (7)
H10A—C10—H10B	109.5	N4—C34—H34A	109.2
C9—C10—H10C	109.5	C33—C34—H34A	109.2
H10A—C10—H10C	109.5	N4—C34—H34B	109.2
H10B—C10—H10C	109.5	C33—C34—H34B	109.2
C7—C11—H11A	109.5	H34A—C34—H34B	107.9
C7—C11—H11B	109.5	N3—C35—N4	104.4 (5)
H11A—C11—H11B	109.5	N3—C35—Pd1	129.7 (5)
C7—C11—H11C	109.5	N4—C35—Pd1	125.9 (5)
H11A—C11—H11C	109.5	C37—C36—N3	107.9 (7)
H11B—C11—H11C	109.5	C37—C36—H36	126.0
C5—C12—H12A	109.5	N3—C36—H36	126.0
C5—C12—H12B	109.5	C36—C37—N4	106.6 (7)
H12A—C12—H12B	109.5	C36—C37—H37	126.7
C5—C12—H12C	109.5	N4—C37—H37	126.7
H12A—C12—H12C	109.5	C43—C38—C39	122.3 (7)
H12B—C12—H12C	109.5	C43—C38—N3	120.9 (7)
N2—C13—C14	109.3 (6)	C39—C38—N3	116.8 (7)
N2—C13—H13A	109.8	C40—C39—C38	117.1 (9)
C14—C13—H13A	109.8	C40—C39—C46	120.6 (8)
N2—C13—H13B	109.8	C38—C39—C46	122.3 (7)
C14—C13—H13B	109.8	C41—C40—C39	122.2 (9)
H13A—C13—H13B	108.3	C41—C40—H40	118.9
O1—C14—C13	107.1 (6)	C39—C40—H40	118.9
O1—C14—H14A	110.3	C40—C41—C42	119.1 (8)
C13—C14—H14A	110.3	C40—C41—C45	120.6 (12)
O1—C14—H14B	110.3	C42—C41—C45	120.1 (12)
C13—C14—H14B	110.3	C41—C42—C43	121.1 (9)
H14A—C14—H14B	108.5	C41—C42—H42	119.5
C20—C15—O1	120.0 (7)	C43—C42—H42	119.5
C20—C15—C16	119.1 (8)	C38—C43—C42	118.2 (8)
O1—C15—C16	120.7 (8)	C38—C43—C44	121.9 (7)
O2—C16—C17	125.7 (9)	C42—C43—C44	119.9 (8)
O2—C16—C15	114.4 (8)	C43—C44—H44A	109.5
C17—C16—C15	119.9 (9)	C43—C44—H44B	109.5
C18—C17—C16	118.7 (10)	H44A—C44—H44B	109.5
C18—C17—H17	120.6	C43—C44—H44C	109.5
C16—C17—H17	120.6	H44A—C44—H44C	109.5
C17—C18—C19	122.3 (10)	H44B—C44—H44C	109.5
C17—C18—H18	118.9	C41—C45—H45A	109.5
C19—C18—H18	118.9	C41—C45—H45B	109.5
C20—C19—C18	118.9 (10)	H45A—C45—H45B	109.5
C20—C19—H19	120.5	C41—C45—H45C	109.5
C18—C19—H19	120.5	H45A—C45—H45C	109.5
C19—C20—C15	121.0 (9)	H45B—C45—H45C	109.5
C19—C20—H20	119.5	C39—C46—H46A	109.5
C15—C20—H20	119.5	C39—C46—H46B	109.5
O2—C21—C22	116.6 (11)	H46A—C46—H46B	109.5
O2—C21—H21A	108.1	C39—C46—H46C	109.5
C22—C21—H21A	108.1	H46A—C46—H46C	109.5
O2—C21—H21B	108.1	H46B—C46—H46C	109.5
C22—C21—H21B	108.1	N5—C47—S1	177.2 (8)
H21A—C21—H21B	107.3	N6—C48—S2	176.9 (8)
